# Phosphodiesterase Type 5 (PDE5) Inhibitors Sensitize Topoisomerase II Inhibitors in Killing Prostate Cancer Through PDE5-Independent Impairment of HR and NHEJ DNA Repair Systems

**DOI:** 10.3389/fonc.2018.00681

**Published:** 2019-01-17

**Authors:** Jo-Fan Chang, Jui-Ling Hsu, Yi-Hua Sheng, Wohn-Jenn Leu, Chia-Chun Yu, She-Hung Chan, Mei-Ling Chan, Lih-Ching Hsu, Shih-Ping Liu, Jih-Hwa Guh

**Affiliations:** ^1^School of Pharmacy, College of Medicine, National Taiwan University, Taipei, Taiwan; ^2^Department of Cosmetic Science, Providence University, Taichung, Taiwan; ^3^Department of Urology, National Taiwan University Hospital and College of Medicine, Taipei, Taiwan

**Keywords:** phosphodiesterase type 5 inhibitor, PDE5-independent, topoisomerase II inhibitor, homologous recombination DNA repair, non-homologous end joining DNA repair

## Abstract

Human castration-resistant prostate cancer (CRPC) is a significant target of clinical research. The use of DNA-damaging agents has a long history in cancer chemotherapy but is limited by their toxicities. The combination with a safer drug can be a strategy in reducing dosage and toxicity while increasing anticancer activity in CRPC treatment. Phosphodiesterase type 5 (PDE5) inhibitors are used to treat erectile dysfunction through the selective inhibition of PDE5 that is responsible for cGMP degradation in the corpus cavernosum. Several studies have reported that PDE5 inhibitors display protective effect against doxorubicin-induced cardiotoxicity. The combinatory treatment of CRPC with doxorubicin and PDE5 inhibitors has been studied accordingly. The data demonstrated that sildenafil or vardenafil (two structure-related PDE5 inhibitors) but not tadalafil (structure-unrelated to sildenafil) sensitized doxorubicin-induced apoptosis in CRPC cells with deteriorating the down-regulation of anti-apoptotic Bcl-2 family members, including Bcl-xL and Mcl-1, and amplifying caspase activation. Homologous recombination (HR) and non-homologous end joining (NHEJ) DNA repair systems were inhibited in the apoptotic sensitization through detection of nuclear foci formation of Rad51 and DNA end-binding of Ku80. PDE5 knockdown to mimic the exposure to PDE5 inhibitors did not reproduce apoptotic sensitization, suggesting a PDE5-independent mechanism. Not only doxorubicin, sildenafil combined with other inhibitors of topoisomerase II but not topoisomerase I also triggered apoptotic sensitization. In conclusion, the data suggest that sildenafil and vardenafil induce PDE5-independent apoptotic sensitization to doxorubicin (or other topoisomerase II inhibitors) through impairment of both HR and NHEJ repair systems that are evident by a decrease of nuclear Rad51 levels and their foci formation in the nucleus, and an inhibition of Ku80 DNA end-binding capability. The combinatory treatment may enable an important strategy for anti-CRPC development.

## Introduction

DNA-damaging agents have a long history of use in cancer chemotherapy. Use of these agents is limited by dose-limiting toxicities and development of drug resistance (1, 2). Doxorubicin is commonly used to treat a wide range of cancers, including hematological malignancies, many types of solid tumors (lung, prostate, breast, bladder and many others) and soft tissue sarcomas (3–5). Doxorubicin interacts with DNA through intercalation and inhibition of macromolecular biosynthesis and inhibition of topoisomerase II, leading to DNA double strand breaks (DSB) of the cells (6). However, the resistance can occur when DNA damage-sensing and repair capacities alter in response to the stresses (7). DSB can be repaired through two major mechanisms: homologous recombination (HR) and non-homologous end joining (NHEJ). HR repairs irradiation-induced two-ended DSBs in G2 phase and predominantly uses a sister chromatid as a template for DSB repair; therefore, HR works only in S and G2 phases. In contrast, NHEJ is the major DSB rejoining process and occurs in all cell cycle phases (8). Since DNA repair is a crucial chemoresistance mechanism, the combination treatments with agents that act through different mechanisms of action may hinder DNA repair capability and improve chemosensitizing effect.

Castration-resistant prostate cancer (CRPC) has a low therapeutic response to conventional chemotherapy. Doxorubicin is an extensively used cancer chemotherapeutic with major side effects such as myelosuppression and cardiotoxicity (1, 2). However, doxorubicin is still an option by injection in combination with other therapeutic drugs for treating CRPC (9, 10), because monotherapy of doxorubicin only displayed modest activity and docetaxel is the most common therapeutic drug to be combined with. The combined chemotherapy induces good synergy and efficient cell death. The rationale of this study was to seek non-chemotherapeutic other than chemotherapeutic drugs for the combined therapy with doxorubicin to reduce possible side effects but remain synergistic anticancer activity.

Sildenafil, vardenafil, and tadalafil are used to treat erectile dysfunction through the selective inhibition of cGMP-specific phosphodiesterase type 5 (PDE5) that is responsible for cGMP degradation in the corpus cavernosum (11). Numerous animal studies have reported that PDE5 inhibitors display protective effect against myocardial injury from ischemia/reperfusion and doxorubicin cardiotoxicity (12–14). The combined therapy of doxorubicin with sildenafil or similar drugs may reduce the probable cardiotoxicity resulted from doxorubicin. Several studies have demonstrated that some PDE5 inhibitors can induce caspase-dependent apoptosis and anti-proliferation in B-cell chronic lymphatic leukemia (13, 15). They can also sensitize certain types of cancer to doxorubicin through multiple mechanisms, including CD95 involved apoptosis, increased endocytosis-mediated cellular uptake and enhanced generation of reactive oxygen species (16–19). Given that PDE5 inhibitors can sensitize doxorubicin-induced apoptosis in CRPC, a clear understanding of the underlying mechanisms is a central goal for the further development of mechanism-based combination therapy. In this study, we have documented the crucial role and the significance of PDE5 inhibitors in apoptotic sensitization. We show the first time that PDE5 inhibitors sildenafil and vardenafil but not tadalafil significantly sensitize topoisomerase II inhibitors in killing CRPC probably through PDE5-independent impairment of HR and NHEJ DNA repair systems. We anticipate that the mechanism study can contribute to better combination in future CRPC therapy.

## Materials and Methods

### Materials

RPMI 1640 medium, fetal bovine serum (FBS), penicillin, streptomycin and 2′,7′ indicating the DNA damage response.-dichlorodihydrofluorescein diacetate (DCFH-DA) were purchased from GIBCO/BRL Life Technologies (Grand Island, NY). Antibodies of PARP-1, Bax, Bcl-2, Bcl-xL, Bak, Mcl-1, Rad51, α-tubulin, DNA-PKcs, anti-mouse and anti-rabbit IgGs were obtained from Santa Cruz Biotechnology, Inc. (Santa Cruz, CA). Antibodies of γ-H2A.X, Bid, caspase-8, cleaved caspase-9, and Ku80 were from Cell Signaling Technologies (Boston, MA). Caspase-3 was from Imgenex, Corp. (San Diego, CA). Antibodies of p-Chk2^Thr68^ and p-DNA-PKcs^Thr2609^ were from Abcam PLC, Inc. (Massachusetts, US). Antibody of RPA32 was from GeneTex Inc. Antibody of PDE5 was from OriGene Technologies, Inc. (Rockville, MD, USA). PDE5 small interfering RNA (siRNA) was from GE Healthcare Dharmacon Inc. (Chicago, USA). All chemical compounds and anticancer drugs were purchased from Sigma-Aldrich (St. Louis, MO, USA).

### Cell Culture

PC-3 and DU-145 were from American Type Culture Collection (Rockville, MD). Cells were cultured in RPMI-1640 medium with 10% FBS (v/v) and penicillin (100 units/ml)/streptomycin (100 μg/ml). Cultures were maintained in a humidified incubator at 37°C in 5% CO_2_/95% air.

### Flow Cytometric Assay of PI Staining

Cells with the indicated treatments were harvested by trypsinization, fixed with 70% (v/v) alcohol at −20°C for 30 min and washed with phosphate-buffered saline (PBS). After centrifugation, cells were resuspended with 0.5 ml PI solution containing Triton X-100 (0.1% v/v), RNase (100 μg/ml), and PI (80 μg/ml). DNA content was analyzed with FACScan and CellQuest software (Becton Dickinson, Mountain View, CA). Ten thousand events were collected per sample and the data were analyzed using ModFit LT version 3.3.

### Anchorage-Dependent Colony Formation Assay

To assess colony formation effect, the cells (100 cells/well) were seeded in a 6-well plate. After a 10-day treatment with the indicated agent, the cell colonies were rinsed with PBS and stained with 0.4% crystal violet/20% methanol and lysed by 50 mM sodium citrate/50% ethanol. The absorbance of the solution was measured at 595 nm wavelengths.

### Western Blotting

Cells were harvested with trypsinization, centrifuged and lysed in 0.1 ml of lysis buffer containing 10 mM Tris-HCl (pH 7.4), 150 mM NaCl, 1 mM EGTA, 1% Triton X-100, 1 mM PMSF, 10 μg/ml leupeptin, 10 μg/ml aprotinin, 50 mM NaF, and 100 μM sodium orthovanadate. Total protein was quantified, mixed with sample buffer and boiled at 90°C for 5 min. Equal amount of protein (30 μg) was separated by electrophoresis in SDS-PAGE, transferred to PVDF membranes and detected with specific antibodies. The immunoreactive proteins after incubation with appropriately labeled secondary antibody were detected with an enhanced chemiluminescence detection kit (GE Healthcare Life Sciences, Buckinghamshire, UK).

### Comet Assays

Cells were pelleted and resuspended in ice-cold PBS. The resuspended cells were mixed with 1.5% low melting point agarose. This mixture was loaded onto a fully frosted slide that had been pre-coated with 0.7% agarose and a coverslip was then applied to the slide. After the gelation of the cell mixture, the coverslip was removed. The slides were then submerged in pre-chilled lysis solution (1% Triton X-100, 2.5 M NaCl, and 10 mM EDTA, pH 10.5) for 30 min at 4°C. After soaking with pre-chilled unwinding and electrophoresis buffer (0.3 N NaOH and 1 mM EDTA) for 30 min, the slides were subjected to electrophoresis for 15 min at 0.5 V/cm (25 mA). After electrophoresis, slides were stained with 1X SYBR Gold (Molecular Probes) and nuclei images were visualized and captured at 200X magnifications with a Zeiss AxioImager A1 fluorescent microscope (Zeiss, Germany) equipped with a CCD camera (Optronics, Goleta, CA). Over one hundred of cells in each sample were scored to calculate the average of comet tail moment (Tail moment = %DNA_tail_ × Length_tail_) using TriTek CometScore^TM^ software.

### DNA Fragmentation Assay

DNA fragmentation was determined using the Cell Death Detection ELISA^PLUS^ kit (Roche, Mannheim, Germany). The assay was based on quantitative *in vitro* determination of cytoplasmic histone-associated DNA fragments (mono-and oligo-nucleosomes) in cells after the induction of cell death. After the treatment, cells were lysed and centrifuged, and the supernatant was used for the detection of nucleosomal DNA fragments according to the manufacturer's protocol.

### siRNA Transfection

PC-3 cells were seeded into a 6-well plate with 30% confluence for each well and grown for 24 h to 50% confluence. Each well was washed twice with PBS and 1 ml of serum-free Opti-MEM (Life Technologies, Ground Island, NY) was added. Aliquots containing control or PDE5 siRNA (a pooled siRNA sequences other than a single sequence) in serum-free Opti-MEM were transfected into cells using Lipofectamine 2000 according to the instructions. After transfection for 5 h, cells were washed twice with PBS and incubated in 10% FBS-containing RPMI-1640 medium for 48 h. The cells were then treated with or without doxorubicin for 48 h, and the level of protein of interest was detected using Western blotting analysis.

### Immunofluorescence Staining of Nuclear Rad51 Foci

PC-3 cells were grown on coverslips placed in a 6-well plate (1.8 × 10^5^ cells/well). All procedures for immunofluorescence staining were conducted at room temperature. Following treatment with doxorubicin and/or sildenafil for 24 and 8 h of cell recovery in drug-free medium, PC-3 cells were washed twice with PBS and fixed with 4% paraformaldehyde in PBS for 20–30 min. After fixation, cells were washed 3 times with PBS, permeabilized with 0.1% Triton X-100 for 10 min followed by 3 times of wash with PBS and then blocked with 5% bovine serum albumin (BSA)/PBS for 1 h. To examine the nuclear Rad51 foci, cells were subsequently stained with the anti-Rad51 antibody (1:200 dilution in 2.5% BSA/PBS) for 1 h with gentle agitation and washed three times with PBS. Cells were next incubated with the FITC-conjugated secondary antibody for 1 h (1:100 dilution in 2.5% BSA/PBS) with gentle agitation. After washing of the cells, nuclear staining was performed using 0.15 μg/ml DAPI for 10 min. Cells on coverslip were finally washed three times with PBS. The air-dried coverslips were next mounted onto glass slides using ProLong® Diamond Antifade Mountant (Thermo Fisher Scientific Inc., Waltham, MA, USA). The slides were then kept in the dark at 4°C for at least one day to dry the antifade mountant. The immunofluorescent images of nuclear Rad51 foci and nuclei were captured at 630X magnifications (63x/1.4NA oil immersion objective lens) using Zeiss AxioImager A1 fluorescent microscope (Zeiss, Germany) equipped with a CCD camera (Optronics, Goleta, CA). At least 100 cells were examined in each sample, and the percentage of cells containing over five Rad51 foci in each sample was estimated.

### DNA End-Binding Activity of Ku80 Protein

Assessment of DNA end-binding activity of Ku80 was carried out using a Ku70/Ku80 DNA Repair kit (Active Motif). In brief, equivalent amounts of nuclear proteins (4 μg) were loaded into an oligonucleotide coated 96-well plate. Then, Ku80 proteins contained in nuclear extract specifically bound to the oligonucleotide. Anti-Ku80 antibody provided by this kit detected DNA bound-Ku80. Addition of the secondary horseradish peroxidase-conjugated antibodies and developing solution provided a colorimetric readout (λ = 450 nm) quantified by spectrophotometry.

### Plasmid-Based NHEJ Assay

The pGL3-control plasmid was completely linearized by HindIII and the linearized DNA was extracted from agarose gel with FavorPrep GEL/PCR purification kit. Cells were co-transfected with linearized DNA and pRL-TK luciferase. NHEJ-mediated ligation was detected by luciferase assay. After treatment, the luciferase activity was assayed by using the Dual-Glo Luciferase Assay System (Promega, Madison, WI, USA). Luciferase activity was normalized using pRL-TK-luciferase activity.

### Data Analysis

Data are presented as mean ± standard error of the mean (SEM) for the indicated number of separate experiments. Student's *t*-test is applied for comparison of two groups. *P* < 0.05 were considered statistically significant.

## Results

### Sildenafil Sensitizes Doxorubicin-Induced Cell Apoptosis in Prostatic Cancer Cells

**Figure 1:**
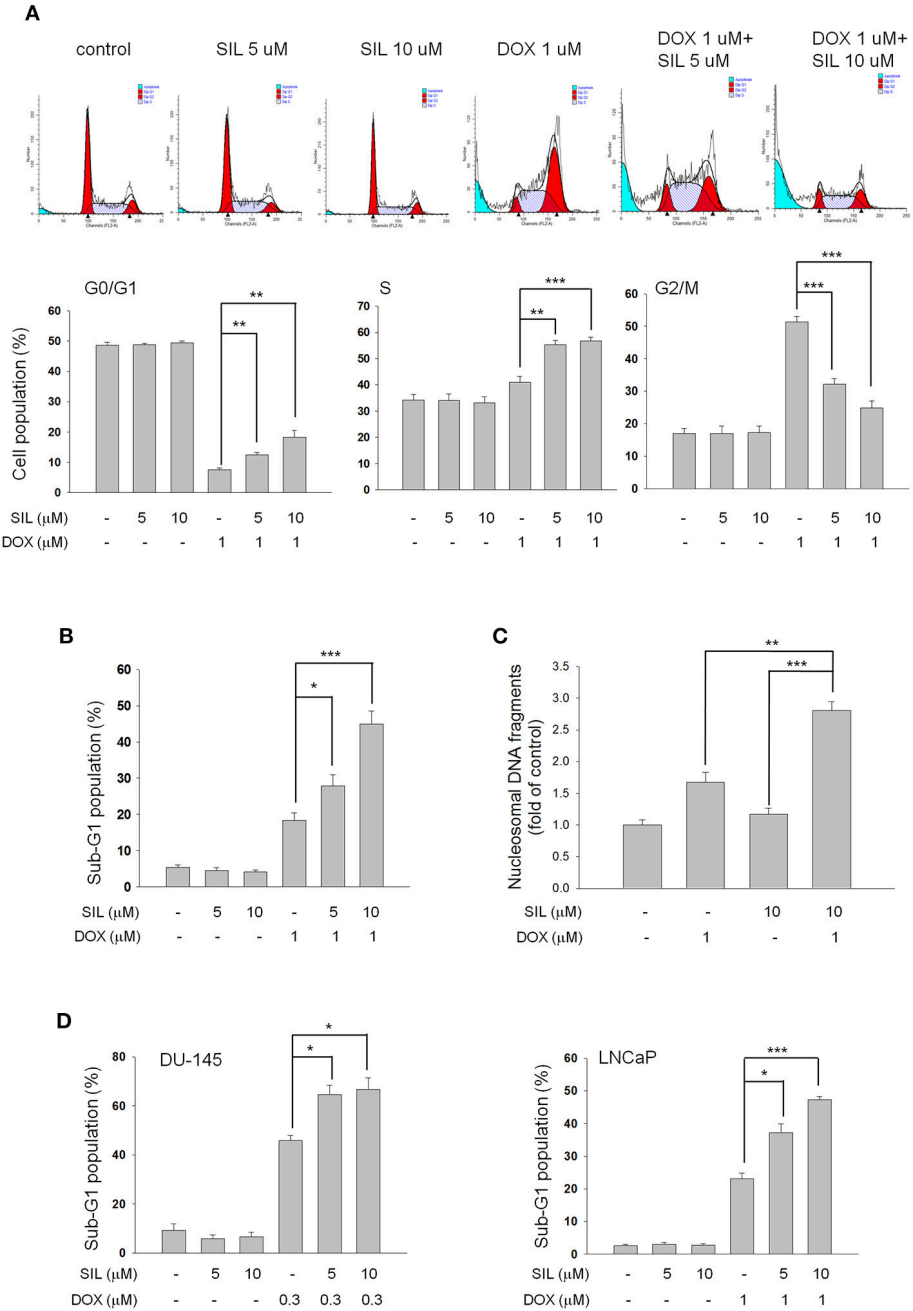
Effect of doxorubicin (DOX) and sildenafil (SIL) on cell-cycle progression and apoptosis. PC-3 **(A–C)**, DU-145 **(D)**, and LNCaP cells **(D)** were incubated in the absence or presence of the indicated agent for 48 h at PC-3 and DU-145 cells or 24 h at LNCaP cells. The cells were fixed with 70% ethanol and stained with propidium iodide to analyze the distribution of cell populations in cell cycle phases by FACScan flow cytometric analysis **(A,B,D)**. PC-3 cell apoptosis was examined by measuring the level of nucleosomal DNA fragments as described in the Materials and methods section **(C)**. Data are expressed as mean ± SEM of three to four independent determinations. ^*^P < 0.05, ^**^P < 0.01, and ^***^P < 0.001.

Sildenafil, vardenafil (two benzenesulfonamide derivatives) and tadalafil (a carboline derivative) are three PDE5 inhibitors. At first, sildenafil was examined in the combination study in PC-3 cells. The data demonstrated that doxorubicin, by itself, induced a significant decrease of cell population at G0/G1 phase associated with an increase at both S and G2/M phases. Sildenafil, by itself, did not modify the cell population in each phase of the cell cycle but partly rescued the decreased population at G0/G1 phase and the increased population at G2/M phase, but further augmented that at S phase (Figure 1A). Besides, it significantly sensitized doxorubicin-induced increase of cell population at sub-G1 phase (Figure 1B). Similar sensitization at the increase of sub-G1 phase population was observed in both DU-145 and LNCaP cells (Figure 1D). Furthermore, sildenafil-induced apoptotic potentiation was substantiated by detection of nucleosomal DNA fragments (Figure 1C) and formation of apoptotic bodies (Supplementary Figure 1).

### Bcl-2 Family of Proteins Plays a Role in Apoptotic Sensitization

**Figure 2:**
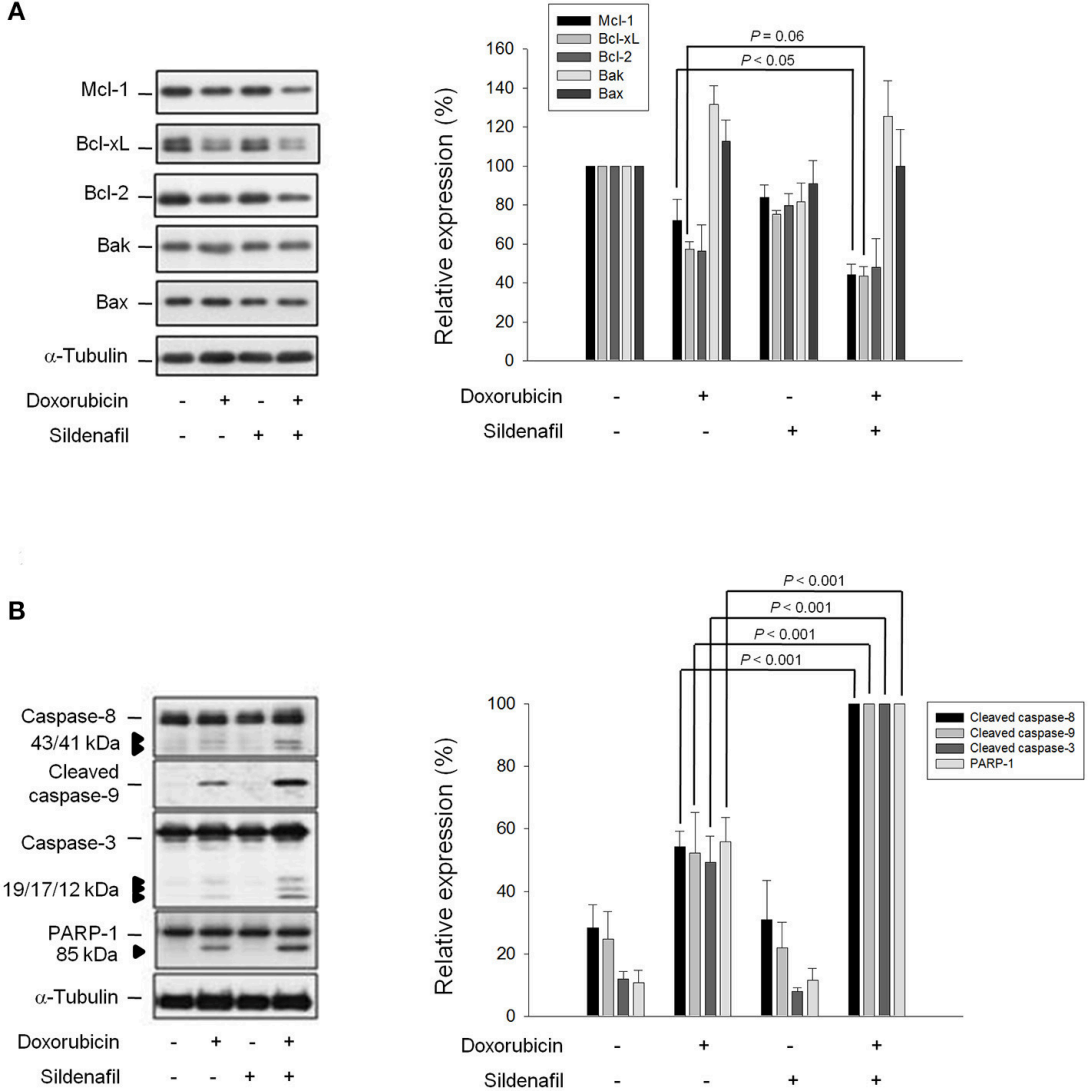
Effect of doxorubicin and sildenafil on the expressions of Bcl-2 family members and caspases. PC-3 cells were incubated in the absence or presence of doxorubicin (1 μM) and/or sildenafil (10 μM) for 24 h. After the treatment, the cells were harvested and lysed for the detection of protein expressions of Bcl-2 family members **(A)** and caspases **(B)** by Western blot analysis. The expression was quantified using the computerized image analysis system Image J software. Data are expressed as mean ± SEM of three to five independent experiments.

Bcl-2 family members that consist of pro-apoptotic (e.g., Bid, Bad, and Bax) and anti-apoptotic members (e.g., Bcl-2, Bcl-xL, and Mcl-1) are a number of evolutionarily conserved proteins and are most notable for the regulation of apoptosis by governing mitochondrial outer membrane permeabilization (20). The expression of Bcl-2 family members is not only closely associated with the androgen-independent phenotype of prostate cancers but also confers an anti-apoptotic capability against androgen withdrawal and cytotoxic chemotherapy (21). Figure 2A demonstrates that sildenafil significantly potentiates doxorubicin-induced down-regulation of anti-apoptotic Bcl-2 family members, including Mcl-1 and Bcl-xL, but did not change the protein expression of Bcl-2 and pro-apoptotic members (Bak and Bax) (Figure 2A). Further identification showed that sildenafil profoundly enhanced doxorubicin-induced activation of caspase −8, −9, and −3 by proteolytic cleavage as well as the increased cleavage of PARP-1, a caspase-3 substrate (Figure 2B).

### Sildenafil Inhibits DNA Repair System but Not Directly Enhances DNA Damage Response to Doxorubicin Action

**Figure 3:**
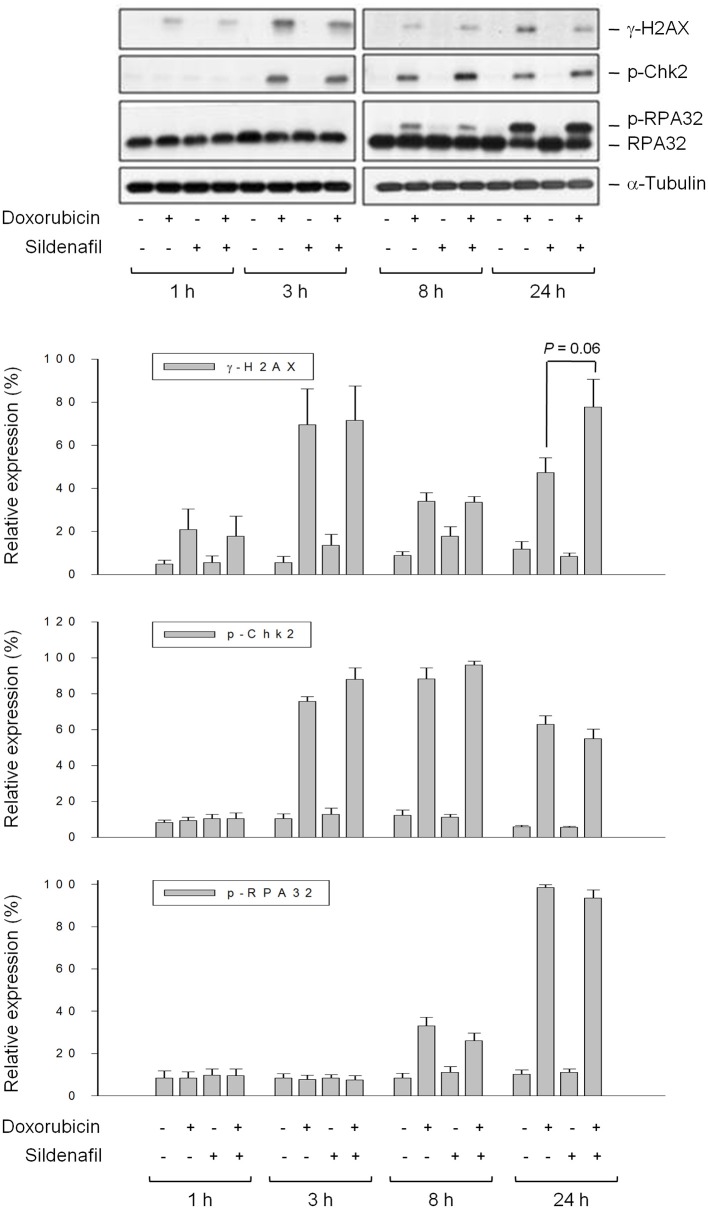
Effect of sildenafil on doxorubicin-induced alterations of several DNA repair proteins. PC-3 cells were incubated in the absence or presence of doxorubicin (1 μM) and/or sildenafil (10 μM) for the indicated time. The cells were harvested and lysed for the detection of protein expression by Western blot analysis. The expression was quantified using the computerized image analysis system Image J software. Data are expressed as mean ± SEM of four independent experiments.

Doxorubicin stabilizes topoisomerase II complex through intercalation and interruption of the DNA chain for replication, preventing the resealing of DNA double helix and thereby blocking the replication process. Accordingly, sildenafil-mediated apoptotic sensitization to doxorubicin can be postulated through the increase of DNA damage reaction and/or the inhibition of DNA repair system. The Single Cell Gel Electrophoresis, also known as a comet assay, was used to detect DNA damage at the level of individual PC-3 cells. Consequently, the short-term treatment (4 h) of cells with doxorubicin resulted in an increase of mean tail moment indicating the DNA damage response. Sildenafil did not sensitize doxorubicin-induced early DNA damage reaction; in contrast, the late DNA damage effect (24-h exposure) was increased by sildenafil (Supplementary Figure 2). It has been widely evident that doxorubicin poisons topoisomerase II-DNA cleavable complexes, leading to the inhibition of religation in the cleaved duplex that induces a DNA DSB. Failure in DNA DSB repair leads to an apoptotic reaction. Although the different time-course data in comet assay could not support the distinction between “real” DNA DSB and apoptotic DNA fragmentation, the sensitization at the 24-h treatment of doxorubicin plus sildenafil group, at least partly, indicated more failed DNA DSB repair and apoptotic response. Several DNA damage-related signals were detected accordingly. Histone H2AX is rapidly phosphorylated (γ-H2AX) in response to DSBs induced by ionizing radiation and a number of cancer chemotherapeutic agents. Time course experiments revealed that the expression of γ-H2AX was increased by doxorubicin after a 3h treatment. Sildenafil did not potentiate the γ-H2AX expression until a 24h treatment (Figure 3). In contrast, sildenafil failed to modify doxorubicin-induced phosphorylation of Chk2 (Figure 3), which regulates a complex network of proteins to elicit DNA repair, cell cycle arrest or apoptosis in response to DNA damage. The phosphorylation of replication protein A (RPA) was also examined. RPA is a trimeric single-stranded DNA-binding complex (RPA70, RPA32, and RPA14) that plays a key role in all aspects of DNA metabolism by stabilizing single-stranded regions of DNA. The data in Figure 3 showed that doxorubicin induced an increase of RPA32 phosphorylation during an 8-h treatment which was blunted by sildenafil although it did not reach a significant level. In contrast, a 24 h doxorubicin exposure caused extensive phosphorylation of RPA32; whereas, sildenafil has little effect on this event (Figure 3). Treuner and the colleagues have reported that extensive phosphorylation of RPA32 is present and participates in apoptotic cell death (22). It was reasonable that the levels of RPA32 phosphorylation were not reduced in the presence of sildenafil for 24h because a potentiation of cell apoptosis was observed at this time course (Figure 3).

### Inhibition of HR and NHEJ Repair Systems Involves in Sensitization Mechanism

**Figure 4:**
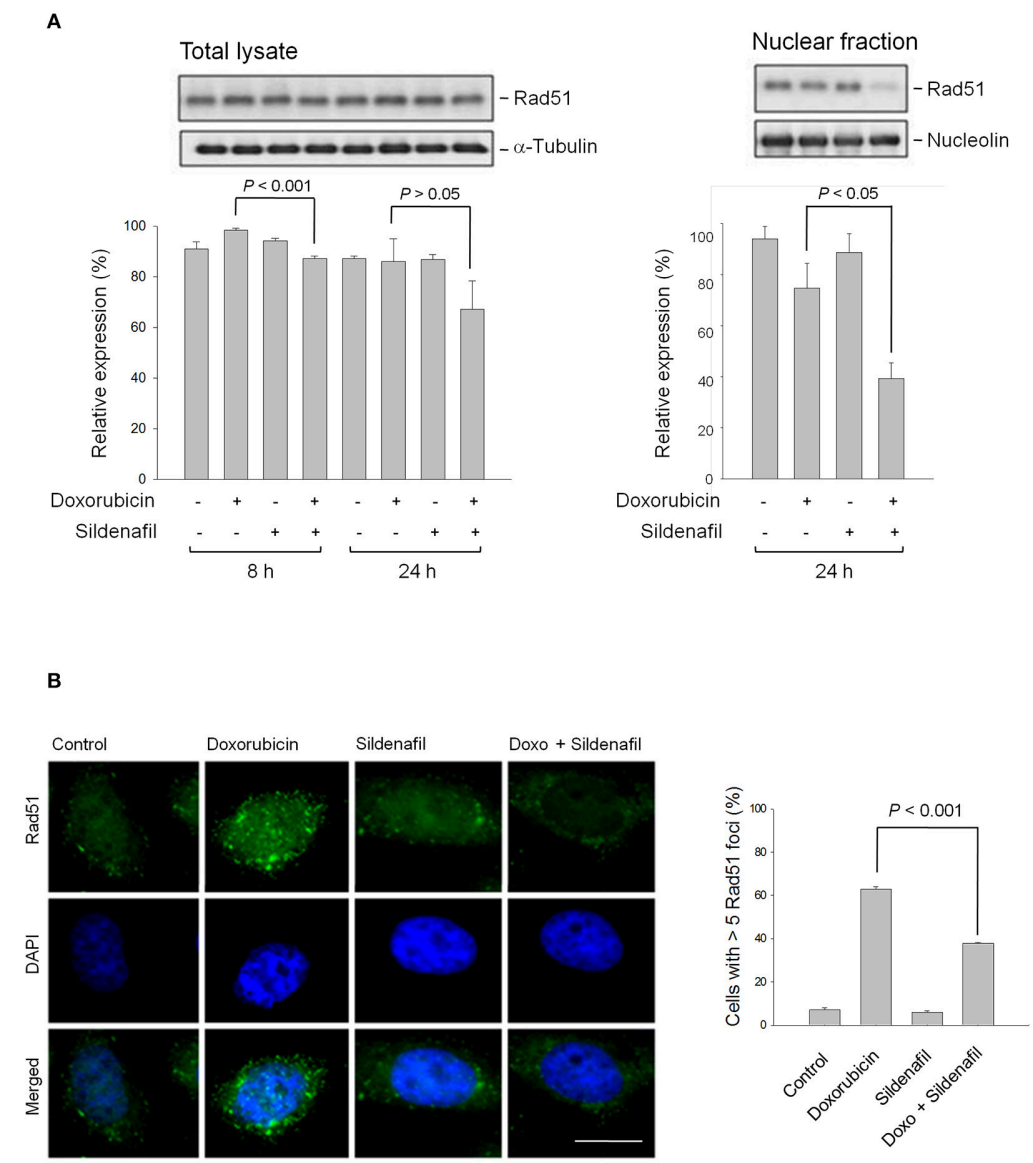
Effect of doxorubicin and/or sildenafil on Rad51 protein expression and cellular distribution. **(A)** PC-3 cells were incubated in the absence or presence of doxorubicin (1 μM) and/or sildenafil (10 μM). The cells were harvested and lysed for the detection of protein expression by Western blot analysis. The expression was quantified using the computerized image analysis system Image J software. Data are expressed as mean ± SEM of three to four independent experiments. **(B)** After a 24-h treatment, the cells were fixed with 4 % paraformaldehyde and the formation of nuclear Rad51 foci was examined using immunofluorescence staining. At least 100 cells were examined in each sample, and the percentage of cells containing more than five Rad51 foci in each sample was estimated. Data are expressed as mean ± SEM of three independent experiments. *Bar*, 20 μm.

DSB repair is not only crucial to genome stability but is also a crucial anticancer target. Cells possess mechanisms that recognize DSB and promote their repair through either HR or NHEJ systems. Rad51 protein plays a central role in HR repair pathway, catalyzing strand transfer between a damaged sequence and its undamaged homolog to permit re-synthesis of the damaged region (23). Rad51 redistribution to chromatin and nuclear foci formation induced by DSBs and interstrand crosslinks are crucial to HR repair. The data demonstrated that the combination of doxorubicin and sildenafil slightly modified the total Rad51 protein expressions but significantly decreased their nuclear levels (Figure 4A). Notably, the nuclear foci formation of Rad51 was dramatically enhanced by doxorubicin, indicating the proceeding of HR repair; however, sildenafil profoundly decreased doxorubicin-induced effects (Figure 4B). In contrast to HR pathway, Ku is a dimeric protein complex which binds to DSB ends and is essential for NHEJ repair pathway (24). Our data showed that the combinatory treatment of doxorubicin and sildenafil did not modify Ku80 protein levels but induced a significant decrease of DNA end-binding of Ku80 (Supplementary Figures 3A,B). Further identification using plasmid based NHEJ assay also demonstrated that the combinatory treatment significantly inhibited NHEJ activity caused by doxorubicin alone (Supplementary Figure 3C).

### PDE5 Inhibitors Show Differential Effects on Doxorubicin-Induced Apoptosis and DNA Repair Regulation

**Figure 5:**
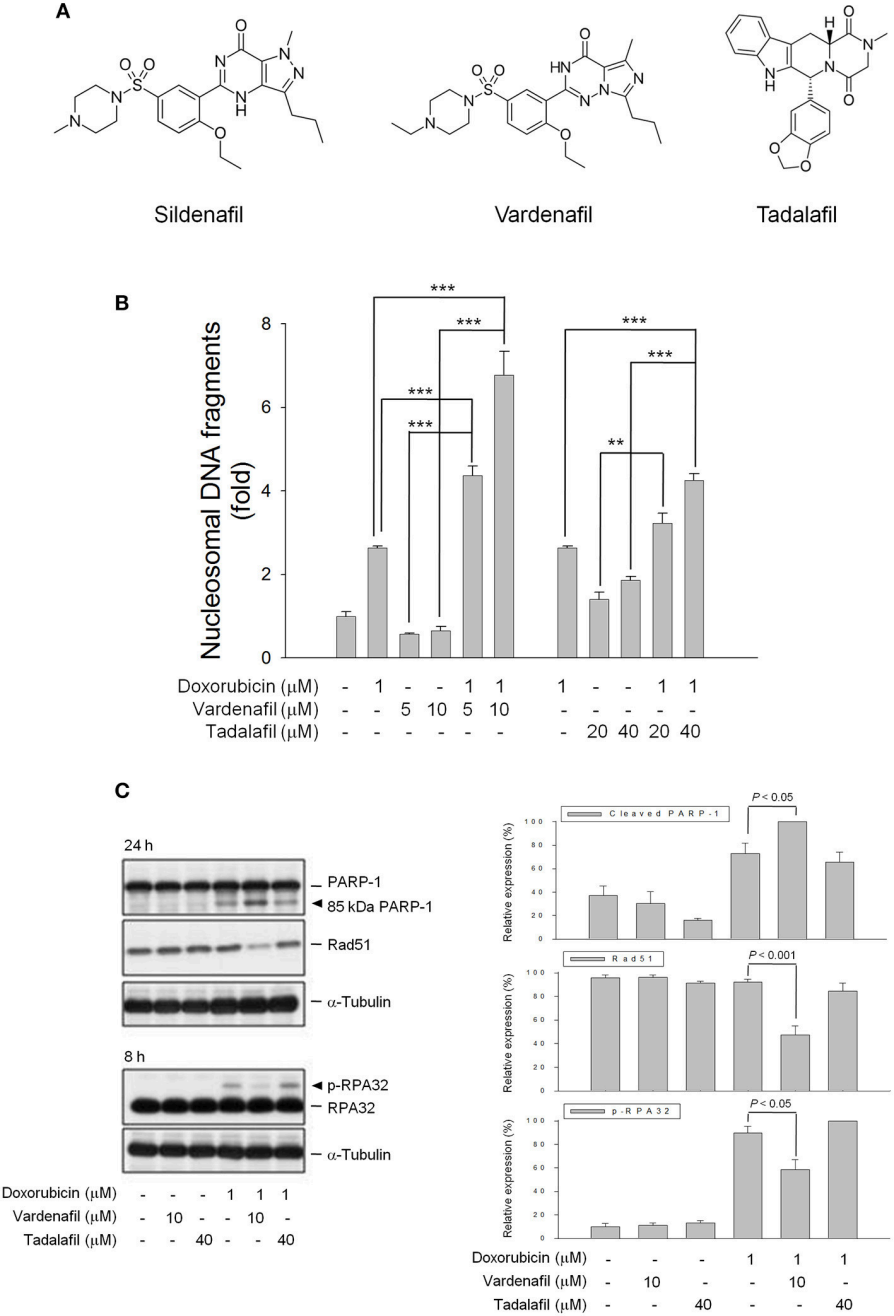
Effect of PDE5 inhibitors on doxorubicin-induced apoptosis and expressions of several proteins. **(A)** Chemical structures of three PDE5 inhibitors. PC-3 cells were incubated in the absence or presence of doxorubicin (1 μM) and/or sildenafil (10 μM) for 24 h **(B)** or for the indicated time **(C)**. Cell apoptosis was examined through measuring the level of nucleosomal DNA fragments as described in the Materials and methods section **(B)** or the cells were harvested and lysed for the detection of protein expression by Western blot analysis **(C)**. The expression was quantified using the computerized image analysis system Image J software. Data are expressed as mean±SEM of three to four independent experiments. ^**^P < 0.01 and ^***^P < 0.001.

Another two PDE5 inhibitors, vardenafil, and tadalafil, were used to examine whether PDE5 inhibitors displayed effects similar to sildenafil action. Vardenafil is a piperazine-containing benzenesulfonamide derivative with high structural similarity to sildenafil. In contrast, tadalafil is a carboline derivative (Figure 5A). Consequently, vardenafil significantly sensitized doxorubicin-induced effect with a synergistic amplification of cell apoptosis (Figure 5B) using synergy quantification of Chou-Talalay method (25). In contrast, only an additive effect was observed in the combinatory use between doxorubicin and tadalafil (Figure 5B). The profound sensitization effects on doxorubicin-induced PARP-1 cleavage, down-regulation of Rad51 protein expression and inhibition of RPA-32 phosphorylation were detected in the presence of vardenafil but not tadalafil (Figure 5C). The data suggest that the sensitization effects are dependent on individual drug other than the inhibition of PDE5.

**Figure 6:**
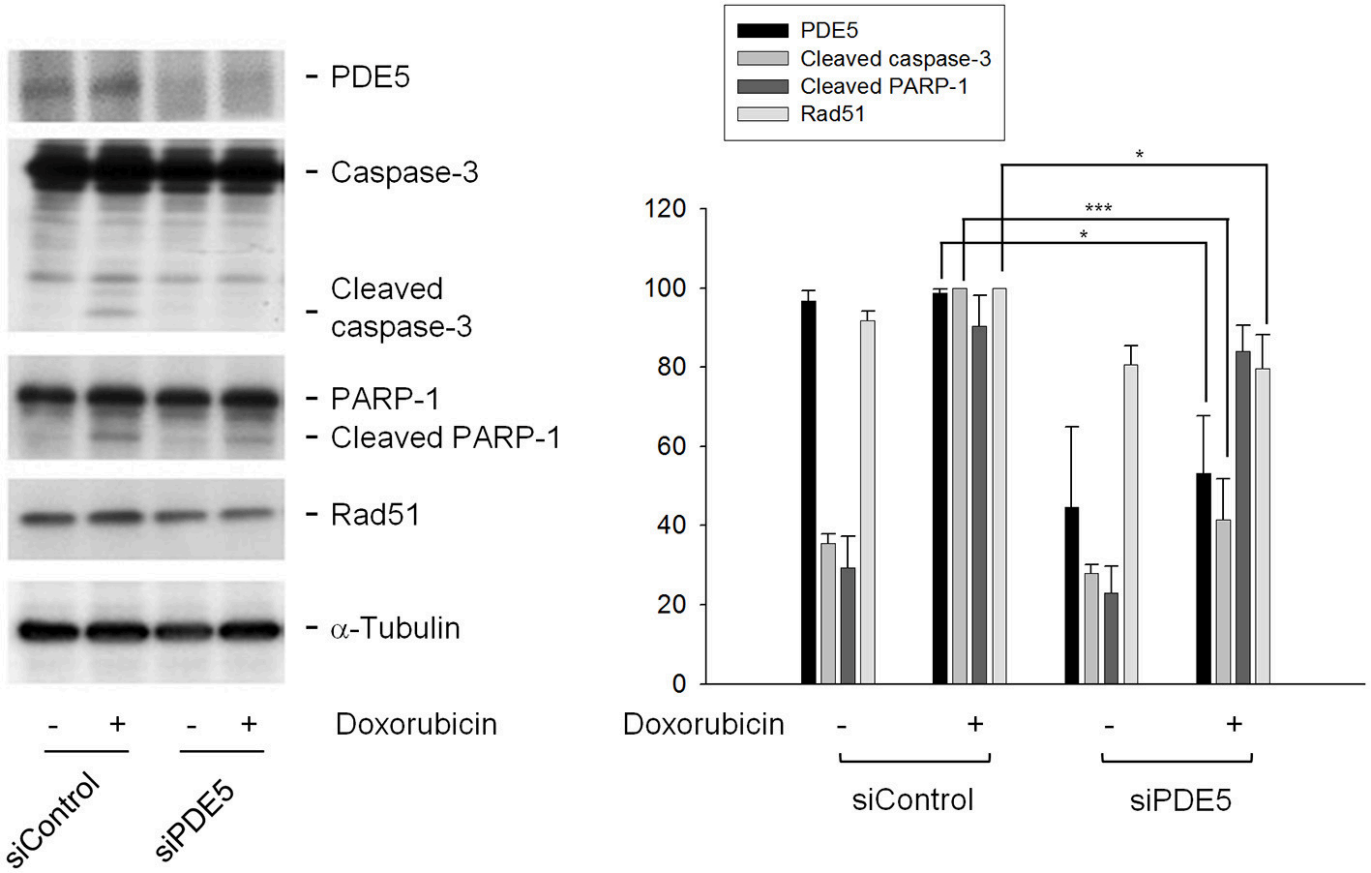
Determination of the functional role of PDE5 on sensitization mechanism. PC-3 cells were transfected with control or PDE5 siRNA. After the transfection, the cells were treated with or without doxorubicin (1 μM) for 24 h and were harvested and lysed for the detection of the indicated protein by Western blot analysis. The expression was quantified using the computerized image analysis system Image J software. Data are expressed as mean ± SEM of three independent experiments. ^*^P < 0.05, ^***^P < 0.001.

To further verify the functional role of PDE5 in the sensitization mechanism, the knockdown of PDE5 expression was performed using siRNA transfection. The data in Figure 6 demonstrated that the protein expression of PDE5 was significantly decreased after the targeted siRNA transfection. However, PDE5 knockdown failed to mimic the presence of sildenafil on inducing the sensitization effects through the detection of cleaved caspase-3 and PARP-1 (Figure 6). The data suggest that PDE5 did not play a crucial role in the sensitization pathway. Notably, Rad51 protein expression was significantly decreased after the exposure of *PDE5*-konckdown cells to doxorubicin, it was probably due to the *PDE5*-konckdown regardless of the presence of doxorubicin (Figure 6).

## Discussion

Researchers have long been interested in the approaches to augment doxorubicin-induced apoptosis in cancer cells by combinatory treatment with non-toxic drugs. Doxorubicin results in the greatest DNA strand breakage in G2/M phase, an intermediate amount in S phase and the least in G1 phase (26). We showed similar data that doxorubicin induced a profound increase at both S and G2/M phases while a decrease at G0/G1 phase. Sildenafil, by itself, did not cause toxicity but significantly sensitized doxorubicin-induced apoptosis associated with a greatest decrease of cell population at G2/M phase, indicating an enhancement of DNA strand breakage in the presence of sildenafil. The release of mitochondrial components into the cytosol and nucleus, and caspase activation are important in regulating apoptotic signals. P53 senses DNA damage to execute mitochondrial dysfunction through up-regulation of pro-apoptotic Bcl-2 family members including Bax and Bak rather than suppression of anti-apoptotic pathways (27). Because PC-3 cells were p53-null, both Bax and Bak expressions were not changed. However, anti-apoptotic members including Bcl-xL and Mcl-1, were decreased after doxorubicin treatment. Sildenafil significantly deteriorated the down-regulation of these Bcl-2 relatives and activation of caspase cascades. These data suggest the involvement of mitochondria-mediated intrinsic apoptotic pathway in the sensitization mechanism.

Topoisomerase II poisons trap topoisomerase II and prevent the enzyme from religating the cleaved DNA, leading to rapid DSBs. Sildenafil did not increase the rapid DNA damage using comet assay probably indicating that sildenafil neither affected doxorubicin-induced stabilization of topoisomerase II-DNA complex nor directly increased DSBs formation. The phosphorylation of histone variant H2AX at Ser 139 (γ-H2AX) serves as a common signal in DNA damage and its repair (28, 29). Doxorubicin induced a rapid formation and a certain extent of decline of γ-H2AX expression. The decrease of γ-H2AX expression after achievement of peak values has been considered the transient DSBs at sites of collapsed replication forks that are subject to repair (30). The second high level of γ-H2AX in the presence of sildenafil represented “stabilized” and non-repaired DSBs responsible for chromosomal aberrations toward cell death program (31).

Chk2 kinase is one of the key components of DNA damage response. Genotoxic stress triggered Chk2 phosphorylation that regulates a variety of proteins in inducing an appropriate cellular response including cell cycle checkpoint activation, apoptosis and DNA repair. This study showed that doxorubicin induced an increase of Chk2 phosphorylation which was not modified by sildenafil. It could be explained that the Chk2 phosphorylation levels were regulated by two opposite events, the decreased DNA repair and increased apoptosis, which compromised in the combination treatment. A similar explanation could also be applied to RPA32 hyperphosphorylation at a 24 h treatment because RPA32 hyperphosphorylation has been implicated in both DNA repair and apoptotic response (22). In contrast, sildenafil diminished RPA32 phosphorylation at an 8h combination treatment although it did not reach a significant level. Since RPA32 phosphorylation plays a critical role in DNA replication and repair by stabilizing single-stranded regions of DNA, sildenafil-mediated decrease of RPA32 phosphorylation might suggest the partly inhibition of repair of single-stranded DNA at an early time (22, 32). However, it needs further identification for clear understanding.

NHEJ and HR are two major mechanisms responsible for DSB repair although they are different in the fidelity and template requirements (32). Studies in monitoring DSB repair show that NHEJ-defective cells have reduced repair in all cell cycle phases during DSB lesions while HR-defective cells have an impairment in S and a substantial defect in late S and G phases, suggesting that NHEJ is a major DSB repair pathway in all phase stages, whereas HR is active in S and G2 phases (9, 32, 33). Sildenafil sensitized the apoptosis with a decrease at G2 population but an increase at both G0/G1 and S phases. The change of cell population at cell cycle phases could hardly tell the dominant impact of sildenafil on NHEJ or HR repair symtem. However, sildenafil-mediated inhibition of doxorubicin-induced nuclear Rad51 protein expression and foci formation might substantiate an impairment of HR repair (23, 34). However, the impairment of NHEJ repair also contributed to the apoptotic sensitization of sildenafil since a decrease of DNA end-binding of Ku80 and an inhibition of NHEJ activity using plasmid based assay were observed in sildenafil-sensitized cells. Several lines of evidence suggest that some cellular effectors may display a dynamic regulation of Rad51 expression in prostate cancers, including p53, Cdk4 and mitogen-activated protein kinase (MAPK) (35–38). These cellular targets have been examined in this study except for p53 because PC-3 is a p53-null cell line. The data demonstrated that doxorubicin-induced protein expressions of phosphorylated Erk but not the others were significantly increased by sildenafil, probably indicating that Erk inversely regulated Rad51 expression (Supplementary Figure 4). Similar results have been reported in several studies. Lian and colleagues have reported that ochratoxin A induces DSBs and Rad51 down-regulation which can be attenuated in the presence of Erk inhibitors (38). Because Rad51 plays a key role in HR repair and increased Rad51 levels are more resistant to DNA damage, the negative regulation of Rad51 expression indicates a pro-apoptotic role of Erk kinase. Similarly, many studies have suggested that depending on the stimulus Erk can mediate anti-proliferation, apoptosis, autophagy and senescence (39). However, the mechanism of regulating Rad51 expression in the combination treatment needs further elucidation. Moreover, it has been suggested that all repair mechanisms are active in proliferating cells in which PARP-1 is in high levels to sustain single-strand break (SSB) repair, base excision repair and HR (40). Besides, PARP-1 repression by certain pharmacological inhibitors may induce accumulation of SSBs, leading to increased sensitivity to anticancer drugs (41). In this study, sildenafil induced the likely cell cycle arrest and the potentiation of PARP-1 cleavage in the presence of doxorubicin (Figure 1A). These effects could at least partly explain the impairment in HR system.

PDE5 knockdown has been performed to recapitulate the effect of PDE5 inhibitor treatment. PDE5 knockdown did not mimic the presence of sildenafil on sensitizing doxorubicin-induced cleavage of caspase-3 and PARP-1, indicating a PDE5-independent sensitization mechanism. The results were validated by the use of tadalafil which inhibited PDE5 activity but did not duplicate sildenafil-mediated apoptotic sensitization and impairment of DNA repair pathway. However, PDE5 knockdown induced a modest decrease of cellular Rad51 protein level. This effect might be somehow controversial result to the PDE5-independent sensitization mechanism. The clear understanding needs an extensive PDE5 knockdown study. Notably, vardenafil which showed high structural similarity to sildenafil could totally reproduce sildenafil-induced sensitization effects. Furthermore, sildenafil induced apoptotic sensitization not only under the exposure to doxorubicin but also to other topoisomerase II inhibitors (e.g., etoposide and mitoxantrone) other than topoisomerase I inhibitors (e.g., camptothecin) (Supplementary Figure 5). The data suggest that drug-induced topoisomerase II-DNA cleavage complexes or the related DNA repair pathways may be a target of sildenafil and vardenafil. Currently, several drugs have been used for the CRPC treatment, including abiraterone and enzalutamide. However, cardiovascular toxicity is one of the major adverse effects for these drugs and, therefore, treatment-related cardiovascular events should be concerned in the patients (42). It has been suggested that PDE5 inhibitors have protective effect against myocardial injury from ischemia/reperfusion and doxorubicin cardiotoxicity (12–14). Therefore, the combination therapy with PDE5 inhibitors may reduce the cardiovascular toxicity induced by the standard medicine. However, further investigation is needed for the combination study.

## Conclusions

**Figure 7:**
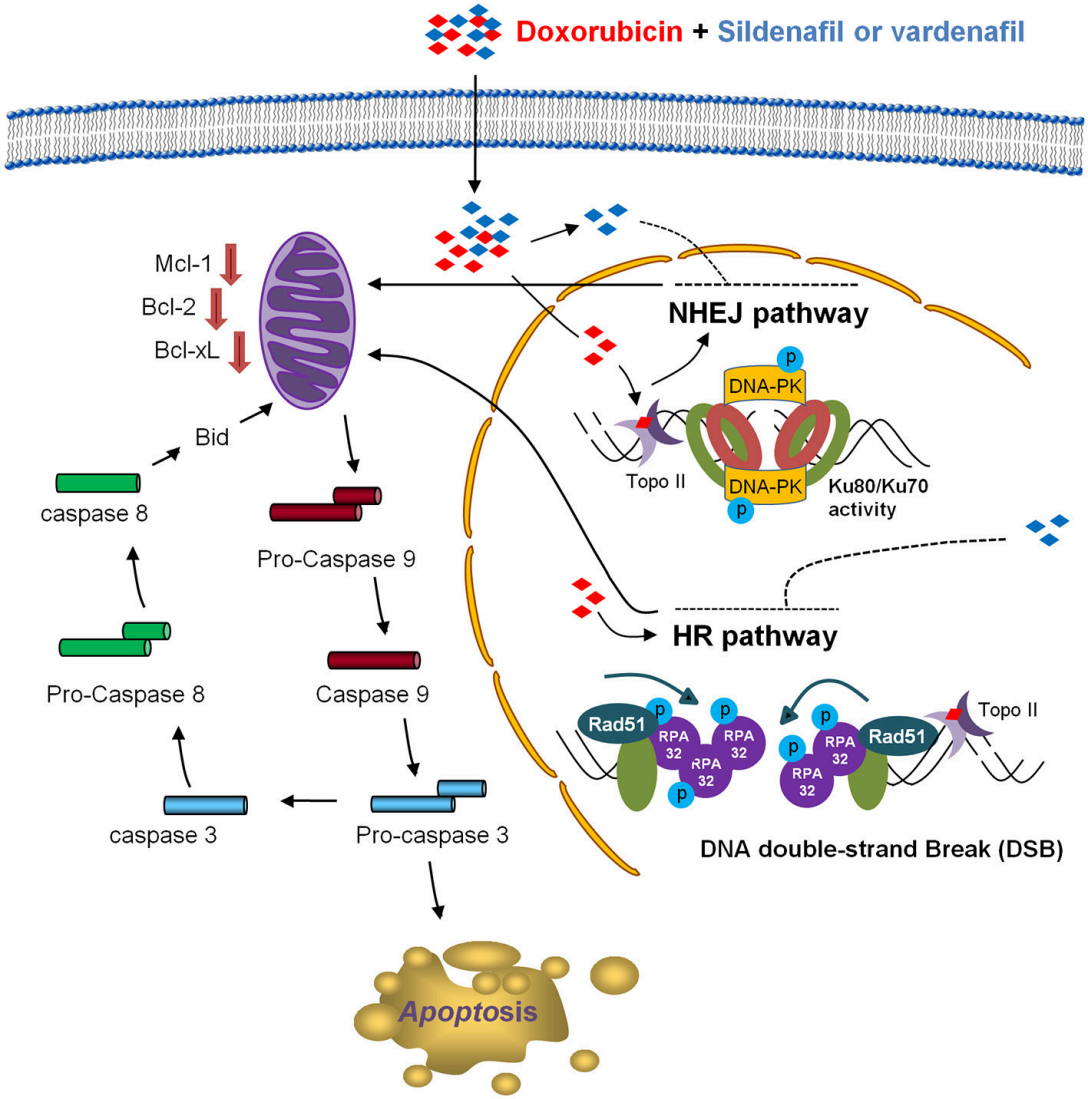
Schematic figure for sildenafil-mediated apoptotic sensitization pathways. Doxorubicin induces cell apoptosis, which is associated with DNA repair processing composed of both HR and NHEJ repair pathways. Sildenafil results in impairment of both repair systems that are evident by Rad51 down-regulation and its decreased foci formation in the nucleus in HR pathway, and a decrease of the DNA end-binding of Ku80 in NHEJ pathway. The combination treatment ultimately causes an enhanced decrease of anti-apoptotic Bcl-2 family members, leading to the activation of caspase cascades and cell apoptosis.

The data suggest that sildenafil and vardenafil, at least partly, induce a PDE5-independent apoptotic sensitization in the presence of doxorubicin in CRPC cells in a sequential manner (Figure 7). Doxorubicin induces cell apoptosis that triggers both HR and NHEJ repair pathways. Sildenafil causes impairment of both repair systems evidenced by a decrease of nuclear Rad51 levels and their foci formation in the nucleus, and an inhibition of Ku80 DNA end-binding capability. The combination treatment deteriorates the decrease of anti-apoptotic Bcl-2 family members, leading to caspase activation and apoptosis. The combinatory treatment between sildenafil (or structure related derivatives) and topoisomerase II poisons may enable an important strategy for anti-CRPC development.

## Author Contributions

S-PL and J-HG contributed to the conception and design of the experiments. J-FC, J-LH, Y-HS, and W-JL performed the experiments and analyzed the data. C-CY, S-HC, M-LC, and L-CH participated the progress reports and troubleshooting in experiments. J-LH and J-HG wrote the manuscript.

### Conflict of Interest Statement

The authors declare that the research was conducted in the absence of any commercial or financial relationships that could be construed as a potential conflict of interest.
