# Supplementary material for: Phosphodiesterase Type 5 (PDE5) Inhibitors Sensitize Topoisomerase II Inhibitors in Killing Prostate Cancer Through PDE5-Independent Impairment of HR and NHEJ DNA Repair Systems
Source: Front Oncol. 2019 Jan 17;8:681. doi: 10.3389/fonc.2018.00681 (PMC6344441; doi:10.3389/fonc.2018.00681)
Supplement: Supplementary file 1 [file Presentation_1.PPTX]

## Slide 1
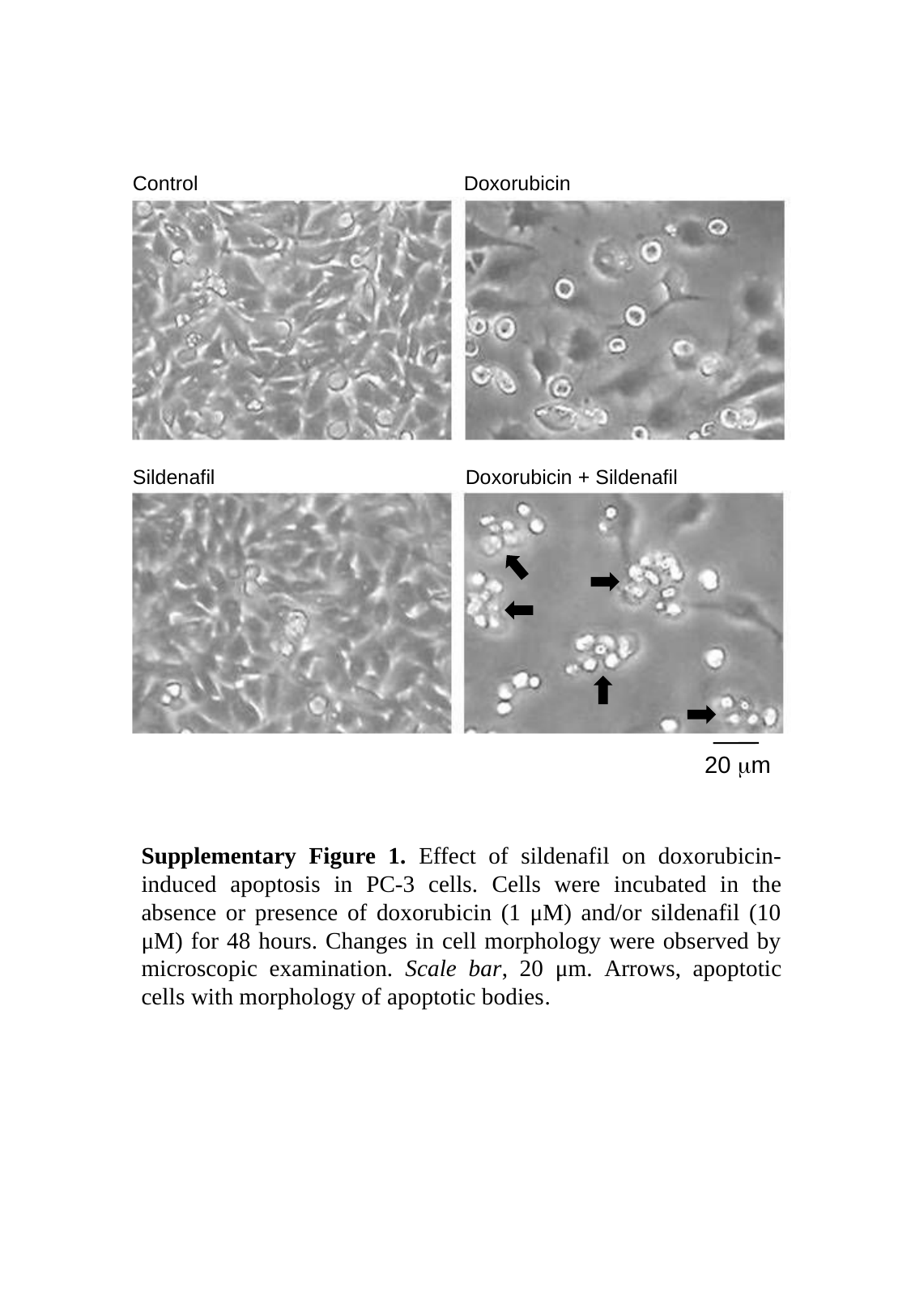

Control
Doxorubicin
Sildenafil
Doxorubicin + Sildenafil
20 mm
Supplementary Figure 1. Effect of sildenafil on doxorubicin-induced apoptosis in PC-3 cells. Cells were incubated in the absence or presence of doxorubicin (1 μM) and/or sildenafil (10 μM) for 48 hours. Changes in cell morphology were observed by microscopic examination. Scale bar, 20 μm. Arrows, apoptotic cells with morphology of apoptotic bodies.

## Slide 2
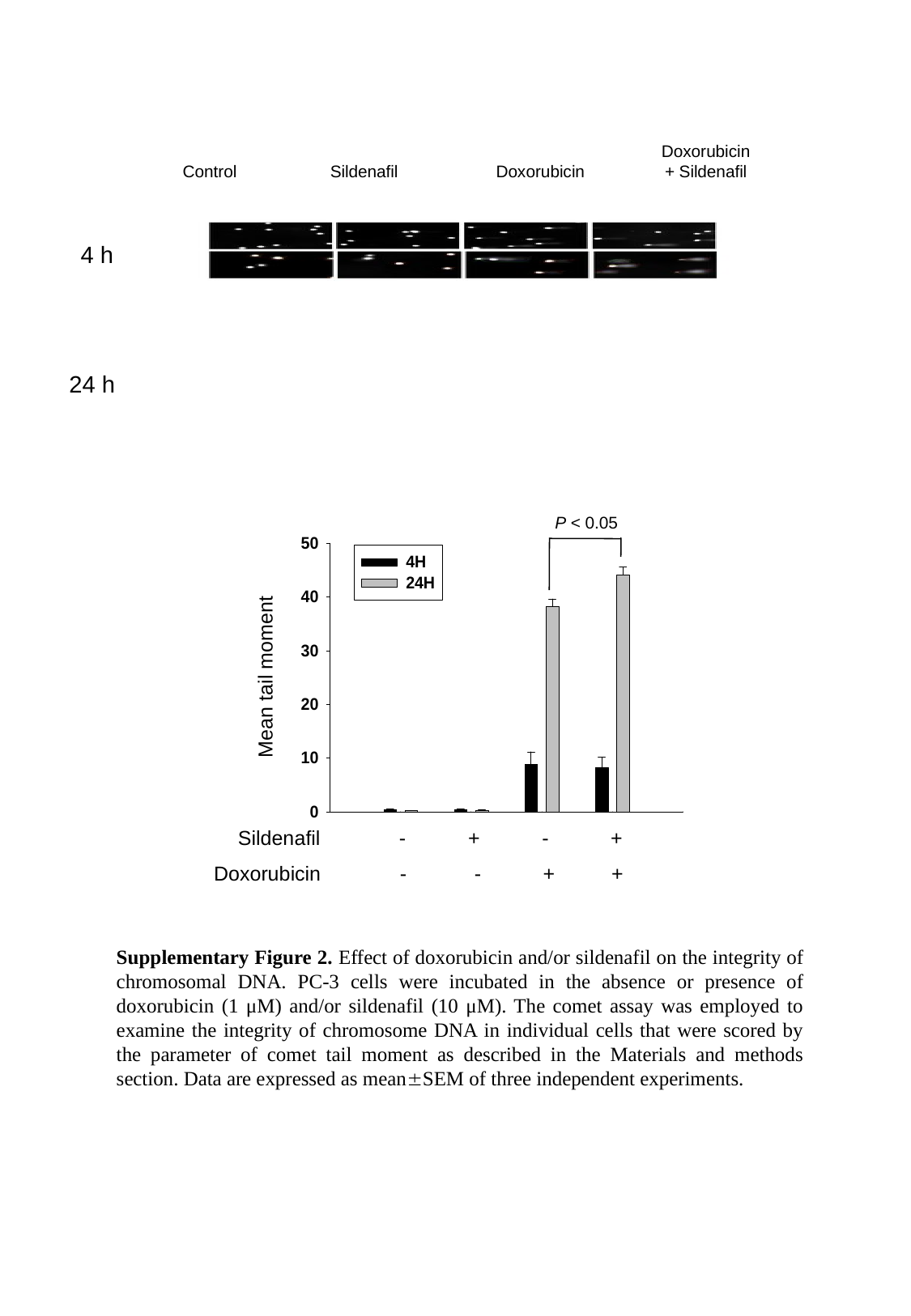

Doxorubicin
+ Sildenafil
Control
Sildenafil
Doxorubicin
4 h
24 h
P < 0.05
Mean tail moment
Sildenafil - + - +
Doxorubicin - - + +
Supplementary Figure 2. Effect of doxorubicin and/or sildenafil on the integrity of chromosomal DNA. PC-3 cells were incubated in the absence or presence of doxorubicin (1 μM) and/or sildenafil (10 μM). The comet assay was employed to examine the integrity of chromosome DNA in individual cells that were scored by the parameter of comet tail moment as described in the Materials and methods section. Data are expressed as mean±SEM of three independent experiments.

## Slide 3
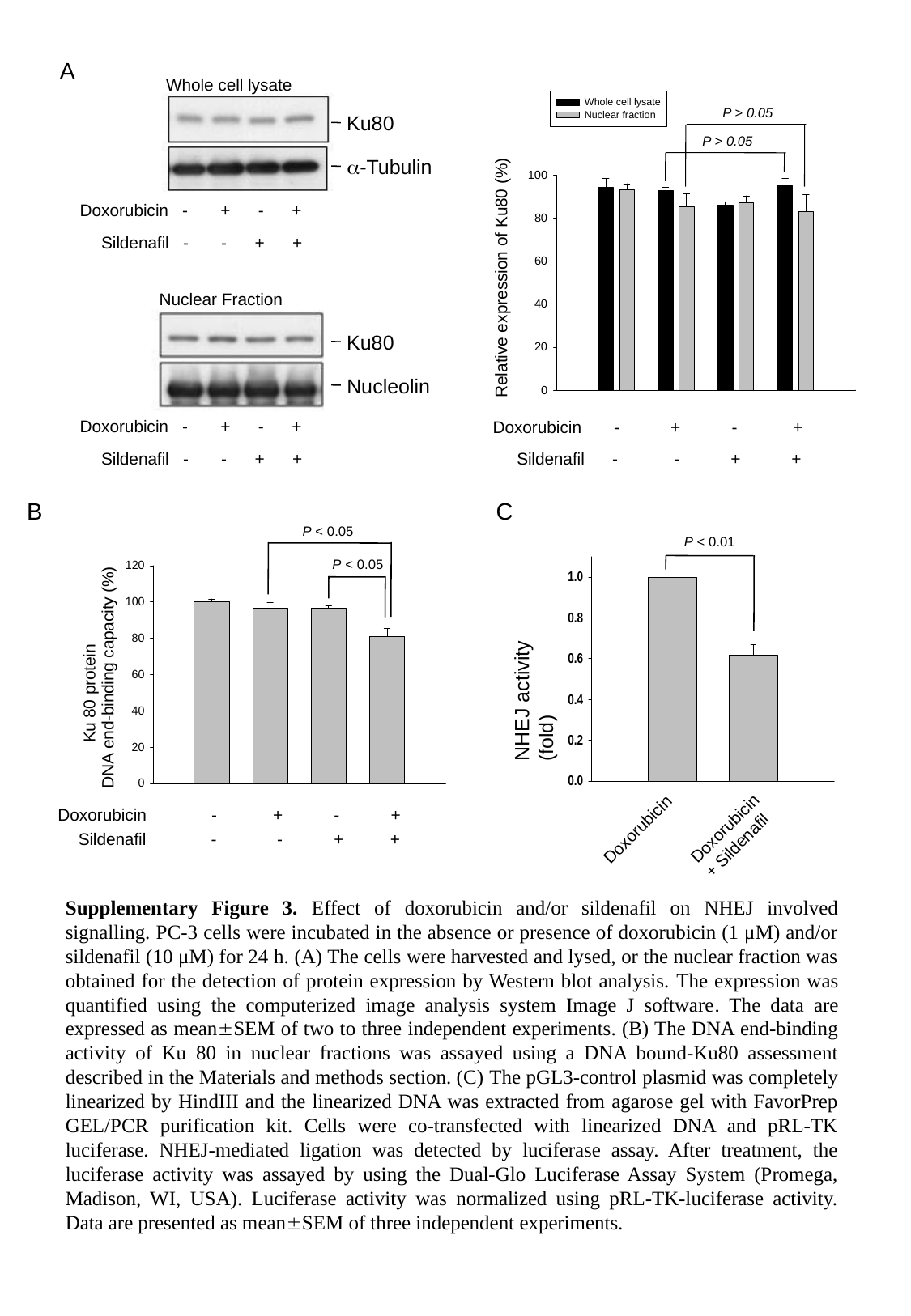

A
Whole cell lysate
P > 0.05
Ku80
P > 0.05
a-Tubulin
Doxorubicin - + - +
Sildenafil - - + +
Relative expression of Ku80 (%)
Nuclear Fraction
Ku80
Nucleolin
Doxorubicin - + - +
Doxorubicin - + - +
Sildenafil - - + +
Sildenafil - - + +
B
C
P < 0.05
P < 0.01
P < 0.05
NHEJ activity (fold)
Doxorubicin - + - +
Doxorubicin
+ Sildenafil
Doxorubicin
Sildenafil - - + +
Supplementary Figure 3. Effect of doxorubicin and/or sildenafil on NHEJ involved signalling. PC-3 cells were incubated in the absence or presence of doxorubicin (1 μM) and/or sildenafil (10 μM) for 24 h. (A) The cells were harvested and lysed, or the nuclear fraction was obtained for the detection of protein expression by Western blot analysis. The expression was quantified using the computerized image analysis system Image J software. The data are expressed as mean±SEM of two to three independent experiments. (B) The DNA end-binding activity of Ku 80 in nuclear fractions was assayed using a DNA bound-Ku80 assessment described in the Materials and methods section. (C) The pGL3-control plasmid was completely linearized by HindIII and the linearized DNA was extracted from agarose gel with FavorPrep GEL/PCR purification kit. Cells were co-transfected with linearized DNA and pRL-TK luciferase. NHEJ-mediated ligation was detected by luciferase assay. After treatment, the luciferase activity was assayed by using the Dual-Glo Luciferase Assay System (Promega, Madison, WI, USA). Luciferase activity was normalized using pRL-TK-luciferase activity. Data are presented as mean±SEM of three independent experiments.

## Slide 4
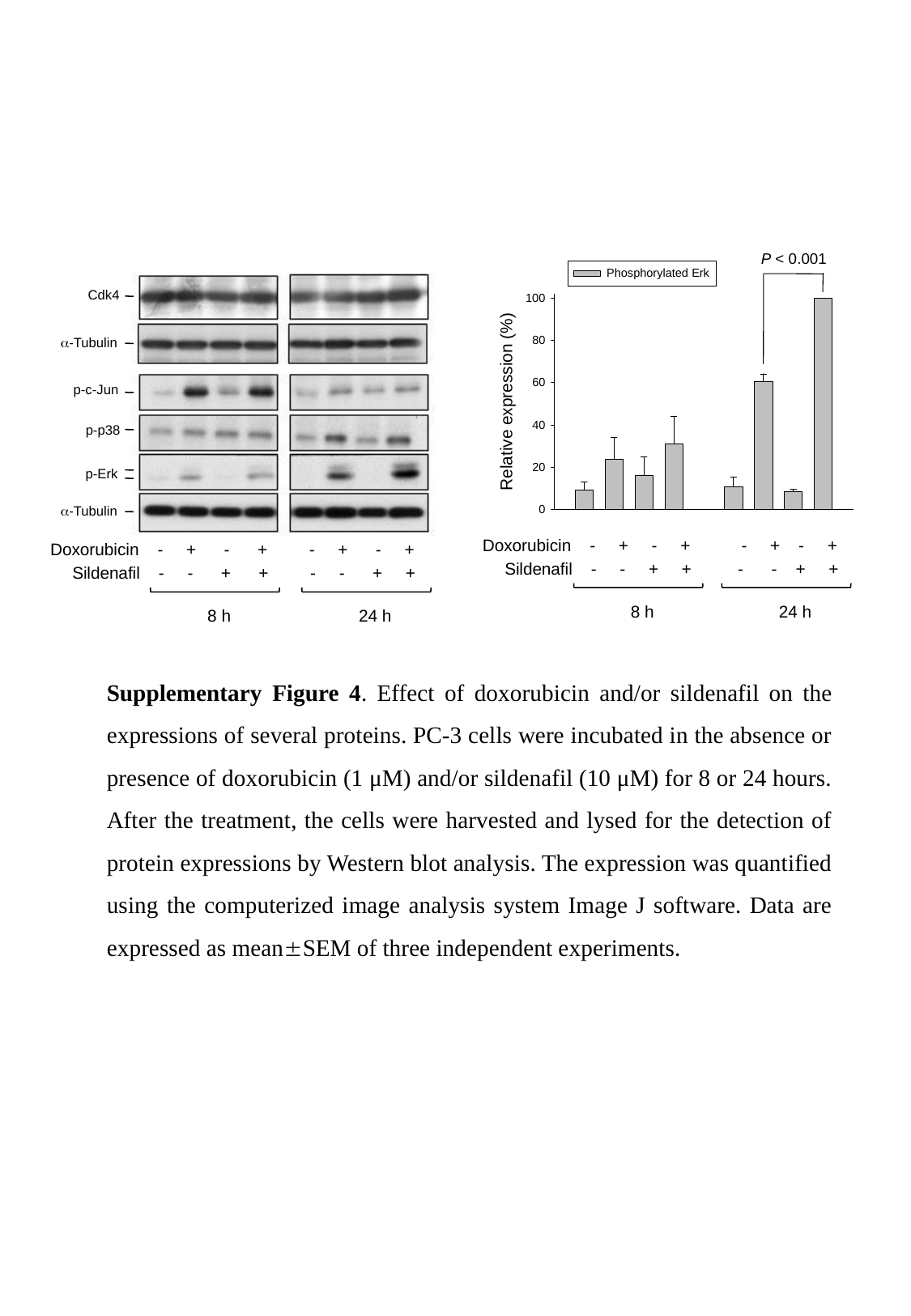

P < 0.001
Relative expression (%)
Doxorubicin - + - + - + - +
Sildenafil - - + + - - + +
8 h
24 h
Cdk4
a-Tubulin
p-c-Jun
p-p38
p-Erk
a-Tubulin
Doxorubicin - + - + - + - +
Sildenafil - - + + - - + +
8 h
24 h
Supplementary Figure 4. Effect of doxorubicin and/or sildenafil on the expressions of several proteins. PC-3 cells were incubated in the absence or presence of doxorubicin (1 μM) and/or sildenafil (10 μM) for 8 or 24 hours. After the treatment, the cells were harvested and lysed for the detection of protein expressions by Western blot analysis. The expression was quantified using the computerized image analysis system Image J software. Data are expressed as mean±SEM of three independent experiments.

## Slide 5
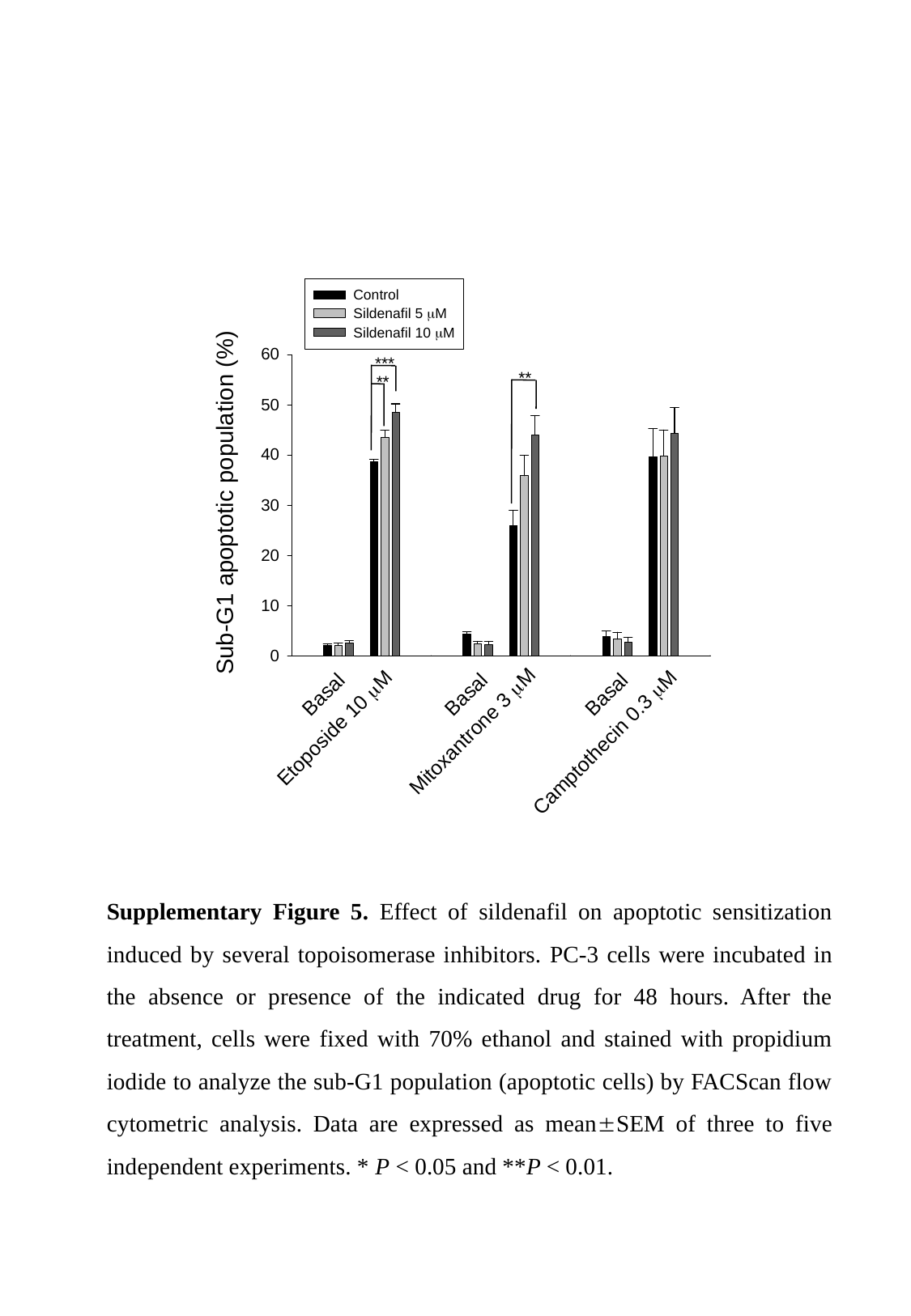

***
**
**
Sub-G1 apoptotic population (%)
Basal
Basal
Basal
Etoposide 10 mM
Mitoxantrone 3 mM
Camptothecin 0.3 mM
Supplementary Figure 5. Effect of sildenafil on apoptotic sensitization induced by several topoisomerase inhibitors. PC-3 cells were incubated in the absence or presence of the indicated drug for 48 hours. After the treatment, cells were fixed with 70% ethanol and stained with propidium iodide to analyze the sub-G1 population (apoptotic cells) by FACScan flow cytometric analysis. Data are expressed as mean±SEM of three to five independent experiments. * P < 0.05 and **P < 0.01.
